# Studies on HDL associated enzymes under experimental hypercholesterolemia: possible modulation on selenium supplementation

**DOI:** 10.1186/1476-511X-8-55

**Published:** 2009-12-16

**Authors:** Harman D Kaur, Mohinder P Bansal

**Affiliations:** 1 Department of Biophysics, Panjab University, Chandigarh 160014, India

## Abstract

**Background:**

Atherosclerosis is a chronic disorder of the arterial wall that starts by formation of fatty streaks and gradually evolves into atherosclerotic plaques. High-density lipoproteins (HDL) blood levels are inversely correlated with atherosclerosis. This beneficial effect of HDL has been partly attributed to its antioxidant properties mediated by paraoxonase1 (PON1) or platelet-activating factor acetylhydrolase (PAF-AH). The present study was aimed to study HDL associated enzymes i.e. PON1 and PAF-AH under experimental hypercholesterolemia and their possible modulation on selenium (Se; an antioxidant) supplementation. Male Sprague Dawley rats were divided into three groups and fed on the control diet, high fat diet (HFD) and HFD + Se respectively for the period of 4 months.

**Results:**

Cholesterol, triglycerides, HDL and LDL levels were significantly increased by HFD feeding. Selenium supplementation lowered the triglyceride level, whereas the other lipid values remained unchanged. Serum selenium levels were reduced by 31% and ROS levels in the liver were 2-fold increased by HFD. Se supplementation, however, diminished the HFD-induced ROS levels by 29%. Furthermore, Se also improved the HFD-mediated reduction of serum PON1 enzyme activity by 34% and PON1 protein levels by 21%. However, no significant effect of Se was detected on the reduced PAF-AH proteins levels in HFD fed rats. mRNA expression of PON1 and PAF-AH in the liver was not affected in the Se treated groups.

**Conclusion:**

Se supplementation appears to be protective in hypercholesterolemia by restoring the antioxidant properties of the HDL associated enzyme i.e. PON1 whereas biological system aims towards maintaining the same PAF-AH levels even on selenium supplementation indicating its probable role in both anti and pro-atherogenic activities. Therefore, Se supplementation might be a valuable approach to limit the adverse effects of hypercholesterolemia and may need further investigations.

## Background

Hypercholesterolemia represents one of the important and recognized risk factor for atherosclerosis [1]. There are compelling evidences indicating the importance of type of fats than the total amount of the fats with respect to the risk of the cardiovascular diseases [2]. Cholesterol is transported within lipoproteins in the blood stream. High density lipoprotein (HDL) cholesterol levels are inversely related to the risk for atherosclerotic events [3] and are found to possess anti-atherogenic activity [4]. Among the risk factors, total cholesterol/HDL cholesterol ratio is considered to be the most predictive for atherosclerosis [5]. The protective effect of the HDL is related partly to enzymes associated with HDL [6-8] and due to its participation in reverse cholesterol transport [9].

Paraoxonase1 (PON1) is one of the enzymes associated with HDL [10]. PON1 was shown to protect against oxidative stress [11,12], a phenomenon that can be attributed to its ability to modulate oxidized lipids in LDL and HDL [13,14], in macrophages [15,16] and also in atherosclerotic plaques [17]. PON is capable of hydrolyzing lipid peroxides in LDL [18]. Serum HDL-associated PON1 reduces oxidative stress in lipoproteins, in macrophages and in atherosclerotic lesion, whereas PON2 acts as an antioxidant at the cellular and not humoral level. The attenuation to atherosclerosis is related to the nutritional anti-oxidative induced increase in HDL-PON activity [19].

PAF-AH is the major enzyme responsible for the catabolism of PAF and PAF like lipids that are also the potent mediators of inflammation [20,21]. Genetic deficiency of PAF-AH in defined human populations increases the severity of atherosclerosis and other syndromes [22]. PAF-AH has marked preference for phospholipids with short chain moieties at syn-2 position and, with the exception of PAF, it can equally hydrolyze oxidized phospholipids containing at syn-2 position polyunsaturated fatty acyl residues [23]. However, during hydrolysis the oxidized phospholipids, PAF-AH liberate the bioactive oxidized free fatty acids [24] and generates lysophopsphatidylcholine, both of which are implicated in the biological actions of ox-LDL [25]. Thus, PAF-AH could play both pro-atherogenic and anti-atherogenic role.

Selenium, an essential trace element, is associated with cardiovascular diseases since years. Selenium deficiency is related to increase in plasma cholesterol levels [26,27], cardiac myopathy [28], other cardiovascular disease and ischemic heart diseases [29,30]. Selenium supplementation leads to decrease in total cholesterol and triglyceride levels [31,32].

Keeping these in view, in the present study influence of selenium was explored on HDL associated enzymes, PON1 and PAF-AH.

## Results

### Selenium levels

**Figure 1:**
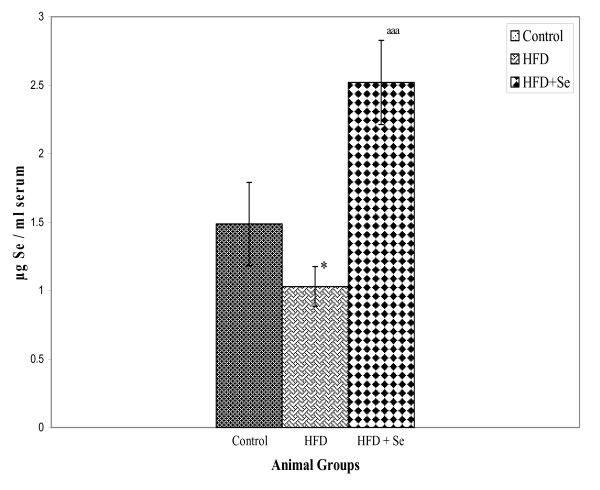
**Serum selenium levels**. *P < 0.05 represents comparison between control and HFD, ^aaa^P < 0.001 represents comparison between HFD and HFD + Se group.

Selenium levels were estimated in the serum of rats from all the groups after 4 months of diet feeding schedule (Figure 1). Significant decrease (P < 0.05) in the Se levels in HFD group was observed in comparison to the control group. However, an apparent increase (P < 0.001) in Se was observed on HFD + Se (1 ppm) supplementation in comparison to the rats fed on only HFD diet.

### Lipid profile

Lipid profile analysis was done in serum (Table 1). Total cholesterol (P < 0.001), triglycerides (P < 0.05) and HDL cholesterol (P < 0.05) levels were found to be significantly increased in HFD group in comparison to control group. A highly significant increase (P < 0.001) was observed in LDL-cholesterol and total cholesterol/HDL-cholesterol ratio in HFD group in comparison to the control group. However, no significant change in various lipid profile parameters except decrease in triglycerides levels was observed in HFD + Se group in comparison to the only HFD fed group.

**Table 1:** Lipid profile in serum after 4 months of Control, HFD and HFD + Se feeding schedule

Lipid profile (mg/dL)	Control	HFD	HFD + Se
**Total cholesterol**	71.79 ± 13.66	451.66 ± 26.73***	438.20 ± 53.91

**Triglycerides**	82.50 ± 6.96	91.98 ± 0.02*	81.26 ± 11.96^a^

**HDL-C**	24.76 ± 4.26	31.70 ± 6.56*	37.58 ± 5.55

**LDL-C**	32.66 ± 2.81	395.52 ± 26.51***	381.00 ± 53.58

**Total cholesterol/HDL-C**	2.90 ± 0.22	14.86 ± 3.86***	11.99 ± 2.95

Data is represented as Mean ± S.D. from 5 independent observations. Data is statistically analyzed by student's t-test. **p < 0.01,*p < 0.05 represent the comparison between control and HFD group. ^a^p < 0.05 represents the comparison between HFD and HFD+ Se group.

### ROS (Reactive oxygen species) levels

**Figure 2:**
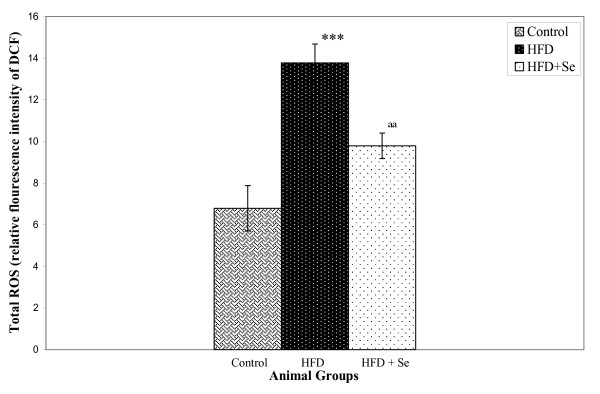
**Reactive oxygen species levels**. ***P < 0.001 represents comparison between control and HFD, ^aa^P < 0.01 represents comparison between HFD and HFD + Se group.

ROS levels in liver homogenates were estimated using a fluorescence probe DCFH-DA. Oxidation of DCFH-DA to DCF was measured as an index of total ROS. A highly significant (P < 0.001) increase in ROS levels was observed in HFD group in comparison to the control group. However, a significant (P < 0.01) decrease in ROS levels were observed in HFD+Se fed group in comparison to HFD fed group (Figure 2).

### PON1 activity

**Figure 3:**
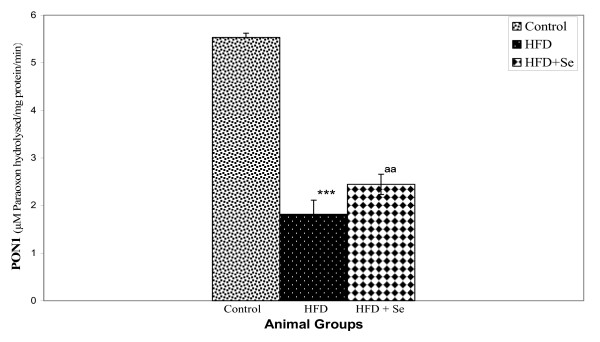
**PON1 enzyme activity**. ***P < 0.001 represents comparison between control and HFD, ^aa^P < 0.01 represents comparison between HFD and HFD + Se group.

PON1 activity was estimated in serum using substrate paraoxon. A highly significant (P < 0.001) decrease in the level of PON1 activity was observed in HFD fed group in comparison to control group. However, a significant (P < 0.01) increase in the level of PON1 was observed in HFD+Se fed group in comparison to HFD fed group (Figure 3).

### mRNA expression of PON1 and PAF-AH

**Figure 4:**
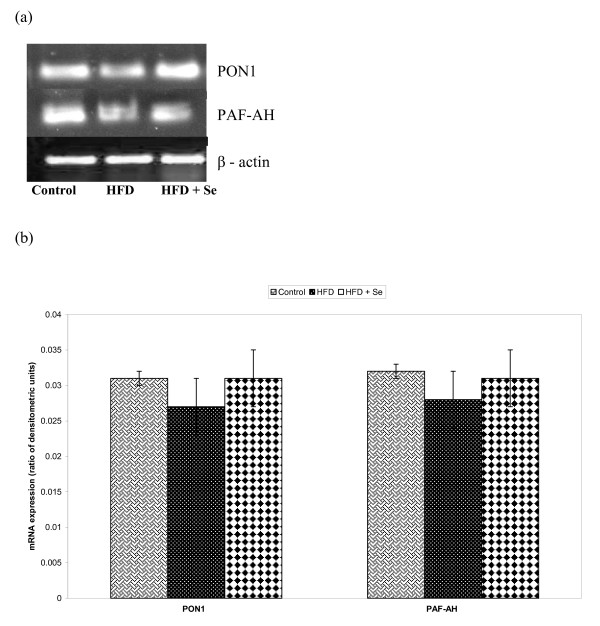
**A - mRNA expression of PON1 and PAF-AH by RT-PCR.** B - Densitometric analysis of PON1 and PAF-AH mRNA expression. Data is represented as Mean ± S.D. from four independent observations. Data is statistically analyzed by student's t-test.

**S**tatistically no significant changes in the mRNA expression of PON1 and PAF-AH were observed in HFD or HFD + Se fed groups in comparison to the control group or HFD fed group respectively Figure 4a, Figure 4b.

### Protein expression of PON1 and PAF-AH by ELISA

PON1 protein levels were significantly (**P < 0.01) decreased in HFD fed group in comparison to control group. However, PON1 levels significantly (*P < 0.05) increased in HFD + Se fed group in comparison to HFD fed group. On the other hand, PAF-AH protein levels were significantly (P < 0.05) decreased in HFD fed group in comparison to the control group. However, there was no significant change observed in HFD + Se fed group in comparison to the HFD fed group (Table 2).

**Table 2:** PON1 and PAF-AH levels by ELISA after 4 months of control, HFD and HFD + Se feeding schedule

A_405 nm_	Control	HFD	HFD + Se
**PON1**	1.55 ± 0.13	1.06 ± 0.01**	1.28 ± 0.12^a^

**PAF-AH**	0.26 ± 0.01	0.25 ± 0.01*	0.26 ± 0.01

Data is represented as Mean ± S.D. from 5 independent observations. Data is statistically analyzed by student's t-test. **p < 0.01,*p < 0.05 represent the comparison between control and HFD group. ^a^p < 0.05 represents the comparison between HFD and HFD+ Se group.

## Discussion

Hypercholesterolemia represents one of the very important and recognized risk factor for atherosclerosis [33]. Abnormally high cholesterol levels (high concentration of LDL and low concentration of HDL) are strongly associated with the cardiovascular diseases. High cholesterol diet leads to the cholesterol deposition in the arterial walls [34]. Compelling evidences indicate the importance of the type of fats than the total amount of the fats with respect to the risk of the cardiovascular diseases [35]. Controlled clinical trials have shown that replacing saturated fats with unsaturated is more effective in lowering serum cholesterol and reducing the risk of cardiovascular diseases than simply reducing total fat consumption [36].

Selenium, an essential trace element is proved to be protective against cardiovascular diseases [37]. In the earlier experiments in the authors laboratory, it was found that selenium supplementation at 1 ppm level along with high fat diet (HFD) feeding in rats inhibited the incidence of atherosclerosis as studied by Scanning electron microscopy (SEM) of aorta [38]. In the present study, interestingly the serum selenium levels decreased in HFD group in comparison to the control group. On the other hand, selenium level, as expected, increased when we gave external supplementation of 1 ppm as sodium selenite in HFD + Se fed group.

Excess of lipids in the serum derived from endogenous synthesis/dietary sources initiate atherosclerosis by accumulation in the cells of the arterial wall and provoking atheroma growth [39]. In the present study, total cholesterol and triglycerides levels were found to be significantly increased in HFD group in comparison to control group. Triglyceride levels were found to be greatly reduced in HFD + Se fed group in comparison to HFD fed group. These findings suggest the hypercholesterolemic state as reported earlier [40-42]. The selenium potential against hypercholesterolemia is supported by some other research groups as well [41,43].

LDL has long been implicated in the development of atherosclerosis [44]. It has also been reported that selenium supplementation protect LDL from oxidation and other atherogenic changes [45,46]. HDL has been found to protect against the oxidation of LDL by metal ions in-vitro [47,48] and by reverse cholesterol transport [49]. Further, LDL and HDL levels were found to be significantly increased in HFD group in comparison to the control group. Increase in HDL levels [41,43] on Se supplementation was also reported earlier. Moreover, total cholesterol/HDL ratio is considered to be most predictive for atherosclerosis [48,50]. In the present study, this ratio was found to be increased in HFD group in comparison to the control group suggesting greatest risk for the clinical events related to hypercholesterolemia in HFD group in comparison to other groups.

Reactive oxygen species (ROS) levels were estimated in liver homogenates and were found to be increased 2-folds in HFD group in comparison to the control group. Se supplementation, however, diminished the HFD-induced ROS levels by 29%. This suggests the presence of oxidative stress in HFD fed group which decreased on 1 ppm selenium supplementation possibly due to the anti-oxidative property of Se. Previously also in the author's laboratory, on analysis of glutathione peroxidase, lipid peroxidation, nitric oxide synthase (NOS) and reduced/oxidized glutathione ratio in aorta, liver and serum, it was demonstrated that the reduced incidence of atherosclerosis on selenium supplementation was due to the anti-oxidant function of selenium [51,52]. Also in-vitro studies, in author's lab, demonstrated the role of mitogen stimulated lymphocytes and macrophage NO production on selenium supplementation in HFD-induced atherogenesis in rats [53].

Work from a number of laboratories have suggested that the protective effect of HDL may be due to the enzymes associated with HDL [6,48,54] i.e PON1 (Paraoxonase 1), LCAT (Lecithin acyl transferase), PAF-AH (platelet activating factor-acetyl hydrolase)

PON1, an HDL associated enzyme, synthesized in liver, was shown to protect against oxidative stress [11,12], a phenomenon that can be attributed to its ability to modulated oxidized lipids in LDL and HDL [13,14], in macrophages [15,16] and also in atherosclerotic lesions [17]. In the present study, biochemically, PON1 enzymes activity was found to be significantly reduced in HFD group in comparison to control group. In addition, protein expression of PON1 by Elisa was also found to be significantly reduced in HFD group in comparison to control group. However, Se supplementation improved the HFD-mediated reduction of serum PON1 enzyme activity by 34% and PON1 protein levels by 21%. This suggests that oxidative stress under hypercholesterolemic state leads to the reduced activity of PON1 whereas on selenium supplementation, levels started retrieving. Interestingly, the PON1 mRNA expression studies revealed that there was no significant change at transcriptional level. This suggests that PON1 may possibly be involved in anti-atherogenic activities at translational levels. In conclusion, Se supplementation appears to be protective in experimental hypercholesterolemia by restoring the antioxidant properties of the HDL associated enzyme, PON1.

PAF-AH is the major enzyme responsible for the catabolism of PAF (platelet activating factor). Hypercholesterolemia and inflammation work as partners in atherogenesis [55,56]. Evidences have suggested that genetic deficiency of PAF-AH in defined human population increases the severity of atherosclerosis and other syndromes [57]. PAF-AH possesses marked preference for hydrolyzing oxidized phospholipids containing at syn-2 position polyunsaturated fatty acyl residues [58]. However, during hydrolysis the oxidized phospholipids, PAF-AH liberate the bioactive oxidized free fatty acids [24] and generates lysophopsphatidylcholine, both of which are implicated in the biological actions of ox-LDL [25]. Thus, PAF-AH could play both atherogenic and anti-atherogenic role. In the present study, PAF-AH protein expression by ELISA was found to be significantly decreased in HFD group in comparison to control group whereas no statistically significant change was observed on selenium supplementation. Interestingly, on the other hand mRNA expression revealed that there was no significant change in PAF-AH at the transcriptional levels. From the present study, it is inferred that PAF-AH possibly participates in both pro and anti-atherogenic activities as here the system aims towards balancing the PAF-AH levels but it may need some further investigations in order to designate the exact role of PAF-AH.

## Conclusion

In conclusion, from the results obtained by the present study, Se supplementation appears to be protective in experimental hypercholesterolemia by restoring the anti-oxidative properties of the HDL associated enzyme PON1. However, findings are inconclusive in determining the role of PAF-AH whether pro or anti-atherogenic in role or both and may need some further investigations. Therefore, Se supplementation might be a valuable approach to limit the adverse effects of hypercholesterolemia.

## Materials and methods

### Chemicals

Sodium selenite (Na_2_SeO_3_), 2, 3-diaminonapthalene and Dichlorofluorescein diacetate (DCFH-DA) were purchased from Sigma-Aldrich (St. Louis, Missouri, USA). TRI-reagent and one step RT-PCR kits were obtained from Molecular research Centre (Inc. Cincinnati, Ohio) and QIAGEN, respectively. Antibodies against PON1 and PAF-AH were obtained from Santa-Cruz Biotechnology, Santa Cruz USA. All other chemicals were of analytical grade and were procured from Indian manufacturers.

### Animals

Male Sprague Dawley rats (100-150 g body weights) were obtained from the Central Animal House, Panjab University, Chandigarh. The animals were kept in plastic cages under the hygienic conditions and were fed on special diets. Before initiating the experiment, the animals were adapted to the laboratory conditions for a week. Necessary approvals were obtained from the Institutional Ethics Committee. The animal care and handling were done according to the guidelines set by the World Health Organization (WHO), Geneva, Switzerland, and the Indian National Science Academy (INSA), New Delhi, India.

### Experimental design

Rats were divided into three groups (five animals each group) for the present study. Group I animals served as controls. These animals were fed on Control Diet. Group II animals were fed on High fat Diet (HFD). Group III animals were fed on HFD with 1 ppm selenium supplementation.

### Diet Preparation

Casein based diets i.e. control diet, HFD was prepared in the laboratory itself according to the composition given by Abraham *et al *[59] Table 3.

**Table 3:** Composition of Control and High Fat diet (HFD):

Component	Control Diet (weight %)	High Fat diet (weight %)
Corn Starch	71.0	61.5

Casein	16.0	16.0

Groundnut Oil	8.0	0.0

Coconut Oil	0.0	15.0

Cholesterol	0.0	2.0

Sodium Cholate	0.0	0.5

Salt Mixture	4.0	4.0

Vitamin mixture	1.0	1.0

Potassium Perchlorate	0.0	25 mg/100 g B.W./rat/24 hrs

To the group III animals i.e. HFD + Se group, 1 ppm selenium was given as sodium selenite and was administered along with the high fat diet. The selenium was so chosen that the level is in excess to adequate levels of selenium (0.2 ppm) but well below the sub toxic limits (2.0 ppm).

### Selenium levels

Selenium levels were estimated in serum by fluorimetric method[60]. The assay is based on the principle that Se content in samples on acid digestion is converted to selenous acid. The reaction between selenous acid and aromatic-o-diamines such as 2, 3-diaminonapathalene leads to the formation of 4, 5- benzopiazselenol which displays brilliant lime-green fluorescence when excited at 366 nm in cyclohexane. Fluorescence emission in cyclohexane extract was read on fluorescence spectrophotometer using 366 nm as excitation and 520 nm as emission wavelengths.

### Lipid profile

Lipid profile analysis was done in serum using colorimetric kits to estimate the total cholesterol, triglycerides, HDL, LDL levels. Total Cholesterol level was estimated using CHOP-PAP based kit (Human Diagnostic Germany). Triglyceride levels were estimated using GPO based kit (Accurex Biomedical India). HDL and LDL cholesterol levels were estimated using (Fortress direct kit) enzymatic kit.

### Reactive oxygen species (ROS) levels

Determination of ROS was based on the modified method of Driver *et al *[61]. Liver homogenates were prepared in ice-cold Locke's buffer (154 mM NaCl, 5.6 mM KCl, 3.6 mM NaHCO_3_, 2 mM CaCl_2, _10 mM d-glucose and 5 mM HEPES pH 7.4). The homogenates were allowed to warm at 21°C for 5 min. The reaction mixture containing 10 μM DCFH-DA and 5 mg tissue/mL was incubated for 15 min at room temperature (21°C). After another 30 min of incubation, the conversion of DCFH to the fluorescent product 2, 7 dichloroflourescein (DCF) was measured using fluorescence spectrophotometer with excitation at 485 nm and emission at 530 nm. Background fluorescence (Conversion of DCFH-DH to DCF in the absence of homogenate) was corrected by inclusion of parallel blanks. The relative fluorescence intensity was taken as the measure of amount of ROS in different groups.

### Measurement of PON1 activity

PON1 activity was assessed in serum by measuring the initial rate of Paraoxon hydrolysis to yield p-Nitrophenol at 412 nm at 25°C. The basal assay mixture included 2 mM Paraoxon, 2 mM CaCl_2 _and 0.5 ml serum in 100 mM Tris/Cl buffer. The extinction coefficient for the reaction is 18290 M^-1 ^cm^-1^. Activity is expressed as μM Paraoxon hydrolyzed/mg protein/min [62].

### RNA isolation and mRNA expression of PON1 and PAF-AH using RT-PCR

Total RNA was isolated from fresh liver using Tri-reagent (Molecular research Centre, Inc Ohio, USA) and the quality of isolated RNA was checked on 1.2% agarose gel electrophoresis. For reverse-transcriptase polymerase chain reaction (RT-PCR), primers for PON1, PAF-AH were designed with the aid of software and β- actin primer was taken from literature. The primer sequence for PON1 was Fav- 5'-TGGCATTGGCATTTCCCTTG-3', Rev- 5'-CAGTAGCTTTCACTCCGGTAA-3' and for PAF-AH Fav- 5'-CTGATGACAAGACCCTCCGTG-3', Rev- 5'-CCGTAACCAGTGTGGTCCGGAT-3' and for β-actin Fav- 5'-AGAGCTATGAGCTGCCTGAC-3', Rev-3'-CTGCATCCTGTCAGCCTACG-5'. After pilot experiments, it was found that PCR products for PON1, PAF-AH were progressively amplified till 35 cycles and hence 35 amplification cycles were performed. The RT-PCR reaction (Qiagen kit) used a template cDNA followed by PCR amplification with Accu Taq DNA polymerase in the same tube. PCR products were analysed by 1.5% agarose gel electrophoresis. Densitometric analysis from six independent observations was done by Image-J software.

### PON1 and PAF-AH protein expression by ELISA

Wells were coated with 5 μg of sample for PON1 and PAF-AH in 100 μl of 0.05 M carbonate buffer (pH 9.6) and kept overnight at 4°C, in a moist chamber. Plates were flicked to remove the unbound antigen solution and wells were blocked with 1% BSA in 0.1 M phosphate buffer saline (pH 7.2) for1 hr at 37°C. Plates were flicked and wells were washed with 200 μl of PBS containing 0.05% (v/v) Tween-20. Wells were then incubated with anti-PON1 and anti-PAF-AH respectively, diluted in PBS (containing 0.05% Tween and 1% BSA) and kept for 2 hr at 37°C. Plates were washed again and incubated with anti-goat secondary antibody (peroxidase labeled) for PON1 (1:1000) and PAF-AH (1:1000) for 2 hr at 37°C. Wells were washed further thrice as described above and color was developed by addition of 2, 2'-azino-di (3-ethyl)-benzothiozolinsulphonic acid reagent and absorbance at 405 nm was measured by ELISA reader.

### Statistical Analysis

Data is represented as mean ± S.D. Statistical analysis of the data was performed by student's T-test.

## Abbreviations

A_260_: Absorption at 260 nm; **A**_412_: Absorption at 412 nm; B.W: Body Weight; LCAT: Lecithin Cholesterol Acyl Transferase; LDL: Low Density Lipoproteins; OD: Optical Density; oxLDL: Oxidized Low Density Lipoprotein; PAF: Platelet activating Factor; PAF-AH: Platelet activating Factor-Acetyl Hydrolase; PON1: Paraoxonase1; Se: Selenium; SRB1: Scavenger receptor B (class) 1(type); VLDL: Very Low Density Lipoproteins.

## Competing interests

The authors declare that they have no competing interests.

## Authors' contributions

MPB designed the study and participated in drafting manuscript and result analysis. HDK carried out all the experimental work, participated in statistical analysis and drafting the manuscript under the guidance of MPB. All authors read and approved the final manuscript.

## References

[B1] Descamps OS Gilbeau JP Luwaert R Heller FR Impact of genetic defects on coronary atherosclerosis in patients' suspects of having Familial Hypercholesterolemia Eur J Clin Invest 2003 33 1 9 10.1046/j.1365-2362.2003.01094.x 12492446

[B2] Seidel C Deufel T Jahreis G Effects of fat-modified dairy products on blood lipids in humans in comparisons with other fats Ann Nutr Metab 2005 49 42 48 10.1159/000084176 15761214

[B3] Gordon DJ Probstfield JL Garrison RJ Neaton JD Castelli WP Knoke JD Jacobs DR Jr Bangdiwala S Tyroler HA High density lipoprotein cholesterol and cardiovascular diseases Circulation 1989 1 8 15 2642759 10.1161/01.cir.79.1.8

[B4] Zuliani G Volpato S Blè A Bandinelli S Corsi AM Lauretani F Paolisso G Fellin R Ferrucci L High interleukin-6 plasma levels are associated with low HDL-C levels in community-dwelling older adults: the Inchianti study Atherosclerosis 2007 192 2 384 90 10.1016/j.atherosclerosis.2006.05.024 16787648 PMC2645783

[B5] Navab M Hama-Levy S Van Lenten BJ Fonarow GC Cardinez CJ Castellani LW Brennan ML Lusis AJ Fogelman AM La Du BN Mildly oxidized LDL induces an increased apolipoproteins J/paraoxanase ratio J Clin Invest 1997 99 2005 19 10.1172/JCI119369 9109446 PMC508026

[B6] Mackness MI Arrol S Durrington PN Paraoxanse prevents accumulation of lipoperoxides in LDL FEBS Lett 1991 286 152 154 10.1016/0014-5793(91)80962-3 1650712

[B7] Stafforini DM Zimmerman GA McIntyre TM Prescott SM The platelet activating factor acetylhydrolase from human plasma prevents oxidation modification of LDL Trans Assoc Am Phys 1993 105 44 63 1309005

[B8] Navab M Hama-Levy S Van Lenten BJ Fonarow GC Cardinez CJ Castellani LW Brennan ML Lusis AJ Fogelman AM La Du BN Mildly oxidized LDL induces an increased apolipoproteins J/paraoxanase ratio J Clin Invest 1997 99 2005 19 10.1172/JCI119369 9109446 PMC508026

[B9] Miller GJ Miller NE Plasma HDL concentration and development of ischemic heart disease Lancet 1975 4 1(7897) 16 9 10.1016/S0140-6736(75)92376-4 46338

[B10] Trudy M Subbanagounder G Berliner JA Blanche PJ Clermont AO Jia Z Oda MN Krauss RM Bielicki JK Altered activities of anti-atherogenic enzymes LCAT, paraoxonase, and platelet activating factor acetylhydrolase in atherosclerosis-susceptible mice J Lipid Res 2002 43 477 485 11893784

[B11] Shih DM Gu L Xia YR Navab M Li WF Hama S Castellani LW Furlong CE Costa LG Fogelman Na AM Lusis AJ Mice lacking serum paraoxonase are susceptible to organophosphate toxicity and atherosclerosis Nature 1998 394 6690 284 7 10.1038/28406 9685159

[B12] Fuhrman B Volkova N Aviram M Oxidative stress increases the expression of the scavenger receptors and the cellular uptake of oxidized low density lipoproteins in macrophages from atherosclerotic mice protective role of antioxidants and of paraoxonase Atherosclerotic 2002 161 2 307 16 10.1016/S0021-9150(01)00646-3 11888513

[B13] Navab M Berliner JA Watson AD Hama SY Territo MC Lusis AJ Shih DM Van Lenten BJ Frank JS Demer LL Edwards PA Fogelman AM The yin and yang of oxidation in the development of the fatty streak. A review based on the 1994 George Lyman Duff Memorial Lecture Arteriosclerosis Thromb Vasc Biol 1996 16 7 831 42 10.1161/01.atv.16.7.831 8673557

[B14] Aviram M Rosenblatt M Bisgaier CL Newton RS PrimoParma SL LaDu BN Paraoxanase inhibit high -density lipoproteins oxidation and preserves its functions. A possible peroxidative role for paraoxanase J Clin Invest 1998 101 8 1581 90 10.1172/JCI1649 9541487 PMC508738

[B15] Rozenberg O Rosenblat M Coleman R Shih DM Aviram M Paraoxanase (PON1) deficiency is associated with the increased macrophage oxidative stress: studies on PON1-knockout mice Free radical Biol Med 2003 34 6 774 84 10.1016/S0891-5849(02)01429-6 12633754

[B16] Rosenberg O Shih DM Aviram M Human serum paraoxanase 1 decreases macrophage cholesterol biosynthesis: possible role for its phospholipases-A2-like activity and lysophosphatidylcholine formation Arteriosclerosis Thromb Vasc Biol 2005 23 3 461 7 10.1161/01.ATV.0000060462.35946.B3 12615663

[B17] Aviram M Hardak Human serum paraoxanase (PON1) Q and R selectively decrease lipid peroxides in human coronary and carotid atherosclerotic lesions; PON1 esterase and peroxidase-like activities Circulation 2000 101 21 2510 17 10831526 10.1161/01.cir.101.21.2510

[B18] Mackness MI Arrol S Durrington PN Paraoxanse prevents accumulation of lipoperoxides in LDL FEBS Lett 1991 286 152 154 10.1016/0014-5793(91)80962-3 1650712

[B19] Rosenblat M Aviram M Paraoxonases role in the prevention of cardiovascular diseases Biofactors 2009 35 1 98 104 10.1002/biof.16 19319852

[B20] Snyder F Platelet -activating factor and its analogs: metabolic pathways and related intracellular process Biochem Biophys Acta 1995 1254 231 24 7857964 10.1016/0005-2760(94)00192-2

[B21] Stafforini DM McIntyre TM Zimmerman GA Prescott SM Platelet activating factor-actylhydrolase J Biol Chem 1997 272 17895 17898 10.1074/jbc.272.29.17895 9218411

[B22] Stafforini DM Biology of platelet-activating factor acetylhydrolase (PAF-AH, lipoprotein associated phospholipase A2) Cardiovasc Drugs Ther 2009 23 1 73 83 10.1007/s10557-008-6133-8 18949548

[B23] Stemler KE Stafforini DM Prescott SM McIntyre TM Human plasma activating factor acetlyhydrolase. Oxidatively fragmented phospholipids as substrates J Biol Chem 1991 266 11095 111003 2040620

[B24] Macphee CH Moores KE Boyd HF Dhanak D Ife RJ Leach CA Leake DS Milliner KJ Patterson RA Suckling KE Tew DG Hickey DM The lipoproteins -associated phospholipases A2, platelet activating factor acetylhydrolase, generates two bioactive products during the oxidation of low density lipoproteins: use of a novel inhibiotor Biochem J 1999 338 479 478 10.1042/0264-6021:3380479 10024526 PMC1220076

[B25] Macphee CH Milliner K Moores K Tew DG The involvement of LDL-associated phospholipases A_2 _in atherogenesis Pharmcol Rev Commun 1996 8 309 315

[B26] Huang K Liu H Chen Z Xu H Role of selenium in cytoprotection against cholesterol oxide induced vascular damage in rats Atherosclerosis 2002 162 137 44 10.1016/S0021-9150(01)00707-9 11947907

[B27] Lee O Moon J Chung Y The relationship between serum selenium levels and lipid profiles in adult women J Nutr Sci Vitaminol (Tokyo) 2003 49 397 404 14974729 10.3177/jnsv.49.397

[B28] Vijaya J Subramanyam G Sukhaveni V Abdul SA Latheef Gupta SR Sadhasivaiah G Salam NM Selenium levels in dilated cardiomyopathy J Indian Med Assoc 2000 98 166 179 11016177

[B29] Beaglehole R Jackson R Watkinson J Scragg R Yee RL Decreased blood selenium and risk of myocardial infarction Int J Epidemiol 1990 19 918 922 10.1093/ije/19.4.918 2084022

[B30] Oster O Prillwitz W Selenium and cardiovascular diseases Biol Trace Ele Res 1990 4 91 103 10.1007/BF02917198 1702669

[B31] Wojicicki J Rosewicka L Barcew-Wiszniewska B Samochowiee L Juwiak S Kadlubowska D Tustanowski S Juzyszyn Z Effects of selenium and Vitamin-E on the development of experimental atherosclerosis in rabbits Atherosclerosis 1991 87 9 16 10.1016/0021-9150(91)90227-T 1670289

[B32] Kang BP Mehta U Bansal MP Hyperlipidemia and Type-I 5'-monodeiodinase activity: regulation by selenium supplementation Ind J Biochem Biophys 2000 7 183 187 10.1385/BTER:77:3:231 11204465

[B33] Descamps OS Gilbeau JP Luwaert R Heller FR Impact of genetic defects on coronary atherosclerosis in patients' suspects of having Familial Hypercholesterolemia Eur J Clin Invest 2003 33 1 9 10.1046/j.1365-2362.2003.01094.x 12492446

[B34] Castro C Campistol JM Baretinno D Andres V Transcriptional profiling of early onset diet-induced atherosclerosis in apolipoproteins-E deficient mice Front Biosci 2005 1 1932 1945 10.2741/1669 15769675

[B35] Seidel C Deufel T Jahreis G Effects of fat-modified dairy products on blood lipids in humans in comparisons with other fats Ann Nutr Metab 2005 49 42 48 10.1159/000084176 15761214

[B36] Hu FB Manson JE Willett WC Types of Dietary fat and risk of coronary heart disease: A critical review J Am Col Nutr 2001 20 5 19 10.1080/07315724.2001.10719008 11293467

[B37] Rayman Margaret P The importance of selenium to human health Lancet 2000 356 233 41 10.1016/S0140-6736(00)02490-9 10963212

[B38] Mehta U Kang BPS Kukreja RS Bansal MP SEM of aorta after high fat diet and selenium supplementation Asia/pacific microscopy and Analysis 2001 22 9 10

[B39] Palmer AM Murphy N Graham A Triglyceride-rich lipoproteins inhibit cholesterol efflux to apo-lipoprotein (apo) A1 from human macrophage foam cells Atherosclerosis 2004 173 27 28 10.1016/j.atherosclerosis.2003.12.001 15177121

[B40] Koul D Kukreja RS Atherogenesis: Preventive action of triflouroperazine Atherosclerosis 1987 64 211 214 10.1016/0021-9150(87)90248-6 3606718

[B41] Wojicicki J Rosewicka L Barcew-Wiszniewska B Samochowiee L Juwiak S Kadlubowska D Tustanowski S Juzyszyn Z Effects of selenium and Vitamin-E on the development of experimental atherosclerosis in rabbits Atherosclerosis 1991 87 9 16 10.1016/0021-9150(91)90227-T 1670289

[B42] Abraham R Kumar NS Kumar GS Sudhakaran PR Kurub PA Synthesis and secretion of Apo-B containing lipoproteins by primary cultures of hepatocytes isolated from rats fed atherogenic diet Atherosclerosis 1993 100 75 83 10.1016/0021-9150(93)90069-7 8318065

[B43] Kang BPS Mehta U Bansal MP Hyperlipidemia and Type-I 5'-monodeiodinase activity: regulation by selenium supplementation Ind J Biochem Biophys 2000 7 183 187 10.1385/BTER:77:3:231 11204465

[B44] Mabuchi H Higashikata T Kawashiri MA Clinical applications of long-term LDL-apheresis on and beyond refractory hypercholesterolemia Transfus Apheresis Sci 2004 30 233 243 10.1016/j.transci.2004.01.006 15172629

[B45] Hussein O Roseblat M Refael G Aviram M Dietary selenium increases cellular glutathione peroxidase activity and reduces the enhanced susceptibility to lipid peroxidation of plasma and low density lipoprotein in kidney transplant recipients Transplantation 1997 63 679 685 10.1097/00007890-199703150-00012 9075838

[B46] Gonca S Ceylan S Yardimoglu M Dalcik H Yumbul Z Kokturk S Filiz S Protective effects of vitamin E and selenium on the renal morphology in rats fed high -cholestreol diet Pathobiology 2000 68 258 263 10.1159/000055935 11493758

[B47] Parthasarathy S Barnett J Fong LG HDL inhibit the oxidative modification of LDL Biochem Biophy Acta 1990 1044 275 283 10.1016/0005-2760(90)90314-n 2344447

[B48] Navab M Hama-Levy S Van Lenten BJ Fonarow GC Cardinez CJ Castellani LW Brennan ML Lusis AJ Fogelman AM La Du BN Mildly oxidized LDL induces an increased apolipoprotein J/paraoxonase ratio J Clin Invest 1997 99 8 2005 19 10.1172/JCI119369 9109446 PMC508026

[B49] Miller GJ Miller NE Plasma HDL concentration and development of ischemic heart disease Lancet 1975 4 1(7897) 16 9 10.1016/S0140-6736(75)92376-4 46338

[B50] Moya DeLa Llera Atger MV Paul JL Fournier N Moatti N Giral P Friday EK Rothblat G A cell culture system for screening human serum for ability to promote cellular cholesterol efflux. Relation between serum components and efflux esterification and transfer Arterioscler Thromb 1994 14 1056 1065 8018660 10.1161/01.atv.14.7.1056

[B51] Kang BPS Bansal MP Mehta U Selenium supplementation and diet induced hypercholesterolemia in the rat: changes in lipid levels, malonyldialdehyde production and the nitric oxide synthetase activity Gen Physiol Biophysics 1998 17 71 78 9675557

[B52] Mehta U kang BPS Bansal G Bansal MP Studies of apoptosis and bcl-2 in experimental atherosclerosis in rabbit and influence of selenium supplementation Gen Physiol Biophysics 2002 21 15 29 12168721

[B53] Kang BPS Mehta U Bansal MP Selenium supplementation protects from high fat diet-induced atherogenesis in rats: role of mitogen stimulated lymphocytes and macrophages NO production Ind J Expt Biol 2001 39 793 797 12018582

[B54] Stafforini DM Zimmerman GA McIntyre TM Prescott SM The platelet activating factor -acetylhydrolase from human plasma prevents oxidative modification of low density lipoproteins Trans Assoc Am Physicians 1992 105 44 63 1309005

[B55] Ross R Atherosclerosis -an inflammatory disease N Engl J Med 1999 340 115 126 10.1056/NEJM199901143400207 9887164

[B56] Steinberg D Atherogenesis in perspective: Hypercholesterolemia and inflammation as partners in crime Nat Med 2002 8 1211 7 10.1038/nm1102-1211 12411947

[B57] Stafforini DM Biology of platelet-activating factor acetylhydrolase (PAF-AH, lipoprotein associated phospholipase A2) Cardiovasc Drugs Ther 2009 23 1 73 83 10.1007/s10557-008-6133-8 18949548

[B58] Stemler KE Stafforini DM Human plasma platelet-activating factor acetylhydrolase. Oxidatively fragmented phospholipids as substrates J Biol Chem 1991 266 11095 11103 2040620

[B59] Abraham R Kumar NS Kumar GS Sudhakaran PR Kurub PA Synthesis and secretion of Apo-B containing lipoproteins by primary cultures of hepatocytes isolated from rats fed atherogenic diet Atherosclerosis 1993 100 75 83 10.1016/0021-9150(93)90069-7 8318065

[B60] Hasunama R Ogawi T Kawanishka Y Flouremetric determination of selenium in nanogram amounts in biological materials using 2,3-diaminonapthalene Anal Biochem 1982 26 242 245 10.1016/0003-2697(82)90510-3 6297332

[B61] Driver AS Kodavanti PR Mundy WR Age related changes in Reactive oxygen Species production in rat brain homogenate Neurotoxicol Teratol 2000 22 2 175 81 10.1016/S0892-0362(99)00069-0 10758346

[B62] Aviram M Rosenblatt M Paraoxanase inhibits high -density lipoproteins oxidation and preserves its functions. A possible peroxidative role for paraoxanase J Clin Invest 1998 101 8 1581 90 10.1172/JCI1649 9541487 PMC508738

